# A Mesoscopic Analytical Model to Predict the Onset of Wrinkling in Plain Woven Preforms under Bias Extension Shear Deformation

**DOI:** 10.3390/ma10101184

**Published:** 2017-10-16

**Authors:** Abbas Hosseini, Masoud Haghi Kashani, Farrokh Sassani, Abbas S. Milani, Frank Ko

**Affiliations:** 1 Department of Mechanical Engineering, University of British Columbia, Vancouver, BC V6T 1Z4, Canada; abbas.hosseini@mech.ubc.ca (A.H.); sassani@mech.ubc.ca (F.S.); 2 Advanced Fibrous Materials Laboratory, University of British Columbia, Vancouver, BC V6T 1Z4, Canada; 3 Composites Research Network-Okanagan Laboratory, School of Engineering, University of British Columbia, Kelowna, BC V1V 1V7, Canada; masoud.haghikashani@ubc.ca

**Keywords:** fabrics/textiles, mechanical properties, analytical modelling, wrinkling initiation

## Abstract

A mesoscopic analytical model of wrinkling of Plain-Woven Composite Preforms (PWCPs) under the bias extension test is presented, based on a new instability analysis. The analysis is aimed to facilitate a better understanding of the nature of wrinkle formation in woven fabrics caused by large in-plane shear, while it accounts for the effect of fabric and process parameters on the onset of wrinkling. To this end, the mechanism of wrinkle formation in PWCPs in mesoscale is simplified and an equivalent structure composed of bars and different types of springs is proposed, mimicking the behavior of a representative PWCP element at the post-locking state. The parameters of this equivalent structure are derived based on geometric and mechanical characteristics of the PWCP. The principle of minimum total potential energy is employed to formluate the model, and experimental validation is carried out to reveal the effectiveness of the derived wrinkling prediction equation.

## 1. Introduction

Taking advantage of the unique combination of light weight and strength, Textile Structural Composites (TSCs) have long been recognized as an attractive material for the applications extending from automotive parts to aircraft structures [1]. Among TSCs, Woven Fabric-Reinforced Composites (WFRCs) have become popular for manufacturing the parts with intricate three-dimensional (3D) shapes. In the manufacturing of WFRCs, the formability of composite part plays a pivotal role and is limited by the drapability of reinforcement phase, i.e., woven preform. The previous research has demonstrated that drapability of woven preforms is constrained by a number of modes of defects, including fiber slippage, wrinkling, in-plane waviness, and yarn bunching. Among these, wrinkle formation is the most critical defect mode [2,3], which is also the focus of the current research. Among different classes of woven fabrics, the Plain-Woven Composite Preforms (PWCPs) have been commonly used in characterization research as well as the reinforcement phase in the WFRCs industry [2,3,4,5,6,7,8,9,10,11].

It is generally agreed that the main deformation mode in the draping of PWCPs is in-plane shear between the warp and weft yarns. If large shear deformation is needed in the course of the process, there would be a limit on the extent of the relative warp/weft angle of PWCPs, owing to anticipated defects such as wrinkling [4,5]. In large shear deformation, woven fabrics continue to be deformed through the meso-level mechanisms elucidated in [6] until the trellis gaps reach the thickness of the interlacing yarn. At this point, locking arises, and further shear of PWCPs is accommodated dominantly through lateral compaction of the yarns [2]. The shear deformation of woven fabric in the post-locking stage will proceed to a certain limit, termed as the ‘wrinkling onset’. This instant is one of the main concerns in the forming of PWCPs.

To analyze the shear behavior and the nature of wrinkle formation—due to large shear—in the woven composite preforms, two experimental characterization methods are widely employed in the literature, namely the Bias Extension Test (BET) and the Picture Frame Test (PFT) [7]. In addition to offering a simpler set-up, the BET is known to be more analogous to real forming process of woven fabrics because of the defects induced during the course of the test (such as yarn lateral slippage, wrinkling, and cross-over rotational slippage) [2,6]. Furthermore, shear within yarns—known as intra-yarn shear—does not occur in the BET, inferring that the yarn width is constant during the test prior to locking point.

A considerable amount of research has been conducted and reported in the literature on determining the onset of fabric wrinkling in woven fabrics [2,3,4,5,6,7,8,9,10,11]. Prodromou and Chen [8] are among the pioneers who recognized the importance of a modeling approach to determine locking angle and the onset of wrinkling in the PWCPs. In their model, the locking angle was defined as the shear angle at which the distance between the warp and weft yarns becomes zero. To develop the approach, they modified the pin-joint theory (PJT), which is solely based on the kinematics of the fabric structure, and included fabric construction parameters such as yarn spacing and yarn size in the analytical calculations. Moreover, they assumed that the onset of wrinkling is coincident with the locking, while the fabric structure undergoes further shear deformation due to lateral compaction of yarns; therefore, it provides more room for shear deformation, and the onset of wrinkling is markedly delayed. In their model, the contribution of the thickness of the interlacing yarn on locking and wrinkling has been overlooked. A detailed critic discussion on this approach has been presented in [6] by the authors.

Lightfoot et al. investigated in-plane waviness and out-of-plane tow wrinkles of the composite preforms in the course of draping process [9]. Through their study, they elucidated the mechanism behind the formation of these two defects. In a study conducted by Launay et al. [10], the effect of tension on yarns was experimentally examined. They demonstrated that the onset of wrinkling is delayed by increasing the tension on yarns. In spite of providing reasonable experimental explanations about the wrinkle formation and the affecting parameters, such as yarn tension, a theoretical interpretation was not presented [9,10].

An energy-based model to predict the wrinkle initiation in the PWCPs has been proposed by Zhu et al. [2,11,12]. The study developed an analytical criterion based on the assumption that wrinkles initiate whenever the energy required for in-plane shear deformation mode becomes greater than that of the out-of-plane deformation mode. This assumption could be challenged given the fact that the comparison was made between two terms of energy that are both internal, and their total always equals the external work done by shear forces.

In view of the extent of the literature available and the wrinkle prediction issues outlined above, the present study is aimed to establish an analytical mesoscopic framework for the prediction of wrinkling in the PWCPs due to large in-plane trellising (BET) deformation. To this end, an explanation about the nature of wrinkle formation—deviation from in-plane deformation to out-of-plane deformation—in the PWCPs is first presented (Section 2). Subsequently, an equivalent structure mimicking the behavior of a representative PWCP element in the post-locking stage is proposed (Section 3). The minimum total potential energy principle is applied to determine the shear angle at which a deviation in deformation mode occurs. The analytical prediction is validated using a BET on plain weave carbon fiber fabric (Section 4).

## 2. Mechanism of Wrinkle Formation in the BET

**Figure 1:**
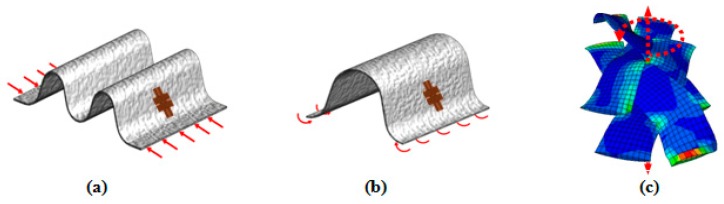
Wrinkles formed due to (**a**) in-plane compression; (**b**) out-of-plane bending and (**c**) in-plane shear.

Wrinkles in the PWCPs can be formed through three distinct loading conditions, namely in-plane compression, out-of-plane bending, and large in-plane shear deformation (Figure 1). Wrinkles induced in the course of the BET belong to the third group [2,3].

**Figure 2:**
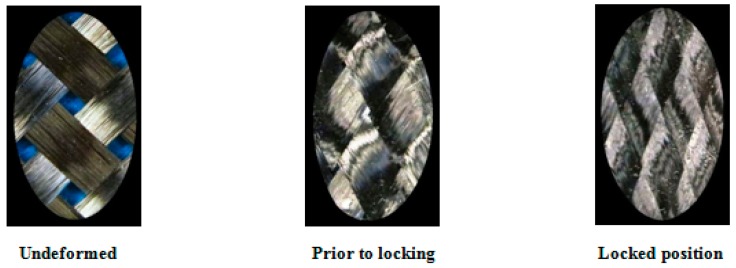
A Plain-Woven Composite Preforms (PWCP) in the course of large in-plane shear deformation.

Based on the pin-joint theory, in the initial stage of shear deformation of PWCPs, yarns rotate with little resistance, which is mainly due to frictional forces between overlapping families of yarns. Afterwards, the shear resistance of PWCPs increases more rapidly when inter-yarn gaps vanish—locking initiates—and adjacent parallel yarns begin to press each other transversely (Figure 2). Further shear deformation of PWCPs at this stage is dominantly by yarns lateral compaction. By growing the shear deformation of PWCPs, the pressure between adjacent parallel yarns rises, triggering the formation of wrinkles.

**Figure 3:**
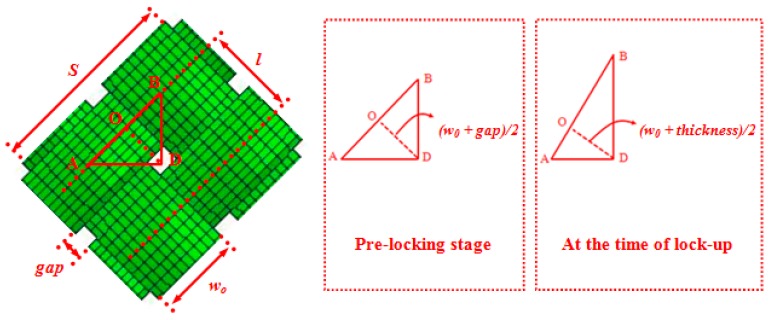
A PWCP representative element at the beginning of shear deformation.

In light of the above discussion, the PWCPs in advance of wrinkling experience two main stages, “pre-locking” and “post-locking”. In a recent research conducted by the authors [6], it is demonstrated that the locking of the PWCPs mainly depends on kinematics and the geometry of the both yarn families in the woven fabric structure: it is defined as an instant at which the distance between the adjacent parallel yarns is bounded by the thickness of the interlacing yarn. Equation (1) is derived for the bias extension test, in which there exists no intra-yarn shear, and is used to predict the locking angle of the PWCPs [6]:

(1) $$\gamma_{Locking}=\cos^{-1}\left(\frac{w_{0}+t}{l}\right)$$

where *γ_locking_*, *w*_0_, *t*, and *l* are the locking angle, initial yarn width, yarn thickness and initial distance between the central axis of the adjacent parallel yarns, respectively (Figure 3).

Contrary to the pre-locking stage, the behavior of PWCPs in the post-locking stage is dominantly governed by the material properties of the yarn (e.g., its transverse compaction resistance and flexural rigidity). Hence, the onset of wrinkling in PWCPs as a whole depends not only on test kinematics and geometry of the fabric elements, but also the material properties of the yarns. Although much earlier research on the behavior of PWCPs in the pre-locking stage has been conducted [6,8], the mechanism of deformation in the post-locking stage has not yet been fully understood.

In other related references [2,13], it was reported that the smallest fabric sub-structure, in which wrinkles can potentially initiate, is the representative element illustrated in Figure 3. Accordingly, this element can be analyzed under large shear angles in the PWCP structures to identify the onset of wrinkling [14].

**Figure 4:**
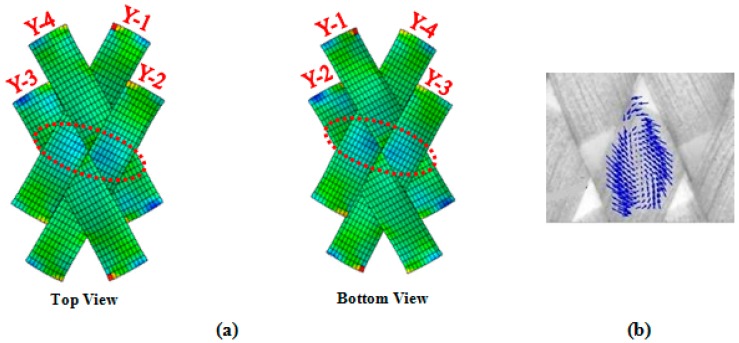
Compressive forces induced between adjacent yarns during in-plane shear deformation of PWCP (**a**) simulation; (**b**) experimental investigation of the yarn strain field in large shear deformation [3].

**Figure 5:**
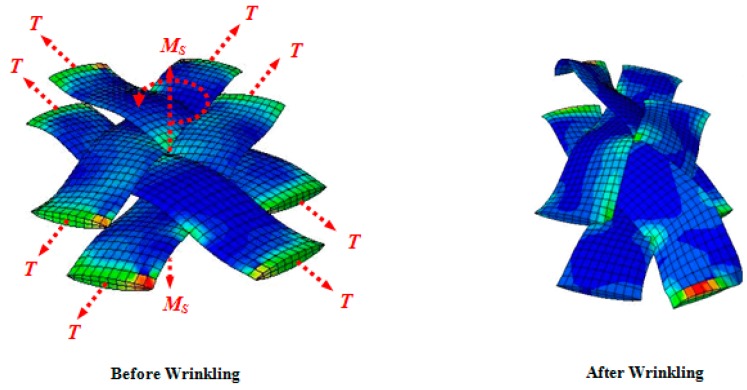
Mechanism of wrinkle formation in a PWCP representative element due to large in-plane shear deformation (*T* and *M_s_* denote the tension along yarn and in-plane shear coupling, respectively).

Figure 4 illustrates the compressive forces established between adjacent parallel yarns during the course of lock-up, and thereof Figure 5 depicts the mechanism of wrinkle formation in the representative element of PWCP.

In mechanical structures under compressive forces, there are discrete values of the load at which secondary equilibrium configurations may appear in the neighborhood of the initial equilibrium position. To put it differently, a system under critical compressive loads admits additional, but adjacent, equilibrium states with different deformation patterns called structural modes [15]. The nature of wrinkling in PWCPs can be analogous to a deviation in deformation mode of the material structure. This analogy opens up a window toward the theoretical analysis of wrinkling phenomenon in PWCPs from an instability point of view.

In the next section, an analogy-based equivalent structure for the representative element of PWCP, which mimics the behavior of the fabric at the post-locking stage, is introduced. Subsequently, the analytical criterion at which deviation of deformation mode from the in-plane to out-of-plane mode becomes possible is derived and validated experimentally.

## 3. Prediction of Fabric Wrinkling Based on an Instability Analysis

### 3.1. Analogy-Based/Equivalent Structure

**Figure 6:**
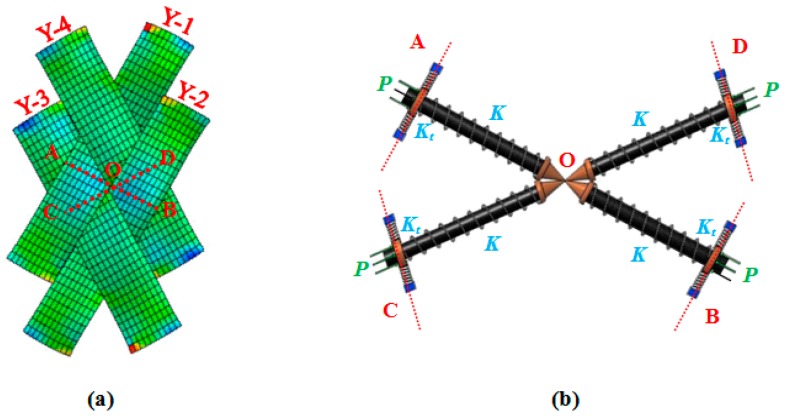
(**a**) Representative PWCP element in post-locking stage; (**b**) four-link equivalent structure mimicking the behavior of a representative PWCP element in post-locking stage.

**Figure 7:**
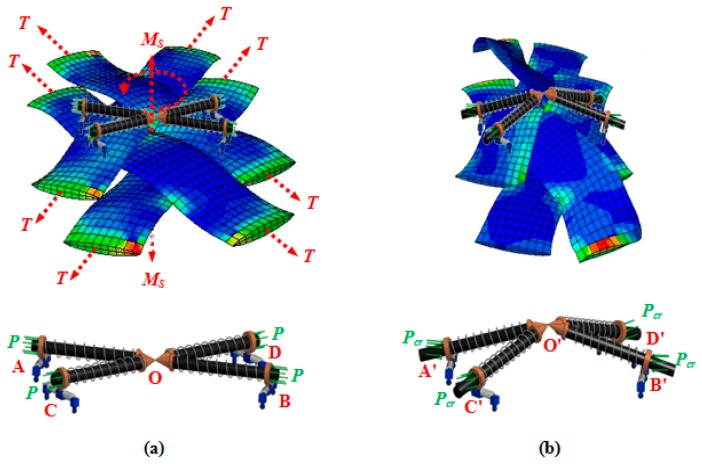
Equivalent structure (**a**) before equilibrium deviation; (**b**) after equilibrium deviation.

As alluded in Figure 4 and Figure 5, the adjacent parallel yarns of the fabric element undergo compressive forces during shear deformation, and subsequently, at a certain instant termed the onset of wrinkling, the fabric structure alters its deformation pattern from the in-plane mode to the out-of-plane mode. Given this definition, a simple four-link equivalent structure mimicking the behavior of a representative PWCP element in the post-locking stage is proposed, as shown in Figure 6. This equivalent structure has been inspired by the phenomenological study conducted by Zhu et al. [16], in which the mechanism of wrinkle formation was experimentally studied and discussed. A mesoscopic finite element simulation was also conducted by the authors (Figure 6 and Figure 7) to observe and identify the nature and mechanism of wrinkling. Based on these mesoscopic trials, the four-link equivalent structure was introduced.

**Figure 8:**
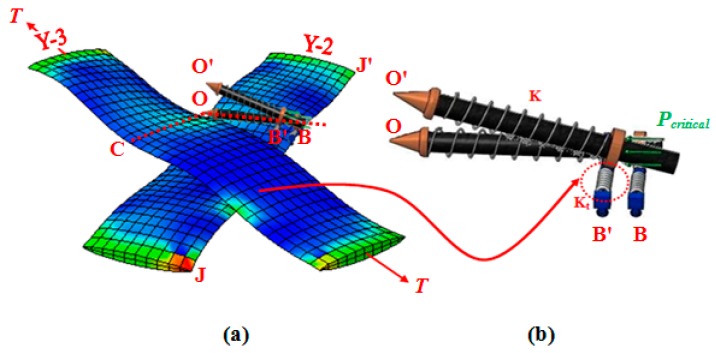
(**a**) The effect of interlacing yarn on wrinkle formation; (**b**) equivalent torsional spring *K_t_*, mimicking the bending effect of interlacing yarn *Y-3* on the out of plane deformation of *Y-2*.

**Figure 9:**
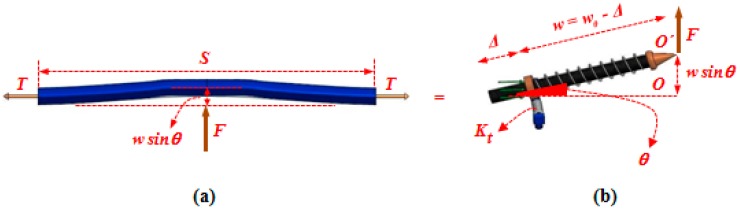
(**a**) Modeling of yarn as a bending beam (made of a linear, transversely isotropic, homogeneous material with an effective flexural rigidity in the longitudinal direction $Q_{b}$); (**b**) an equivalent torsional spring of coefficient *K_t_*.

It is assumed that the instant of deviation in equilibrium mode of this equivalent structure is coincident with the onset of wrinkling in the fabric (Figure 7). This structure, hereafter called PWCP Equivalent (PWCPE), comprises of four rigid bars of length *w*_0_ (initial width of yarns). These bars are clamped elastically at one end against rotation—about the dash-lined axes illustrated in Figure 6—using four torsional springs of stiffness *K_t_*, and linked to ball-joints at the other end (Point **O**). The torsional springs in fact imitate the role of interlacing yarns in the fabric (Figure 8). To elucidate, yarn *Y-2* at the onset of wrinkling tends to rotate about its longitudinal outer edge (i.e., about JJ’ axis in Figure 8) and form a wrinkle. In order to accommodate such deformation, the transverse bending of the interlacing yarn *Y-3* is necessitated, as shown in Figure 9. That is, the interlacing yarn *Y-3* resists against wrinkling. Hence, this out-of-plane resistance of interlacing yarn is modeled by an equivalent torsional spring (*K_t_*). The other yarns in the representative PWCP element simultaneously experience the same sequence of events.

Four sliding springs with the stiffness coefficient *K* are placed around the bars to capture the lateral compaction stiffness of yarns—in the width direction—and their stiffness coefficient needs to be characterized via experimentation (to be discussed in more detail in Section 4.2.2).

The bars are treated as the cores of the sliding springs. The quasi-static compressive forces *P* act on the system, as illustrated in Figure 7. These forces are to model the compaction forces between adjacent parallel yarns in the real fabric. If the compressive forces *P* in the PWCPE structure reach a critical value, termed as *P_cr_*, the equivalent structure will become unstable. At the onset of instability, the PWCPE structure will change its deformation mode from in-plane to out-of-plane (Figure 7). Accordingly, obtaining *P_cr_* is a significant step to predict the onset of wrinkling in the fabric structure. Before that, however, we first need to characterize the stiffness properties of the structure as follows.

#### 3.1.1. Characterization of Stiffness Elements of the PWCPE Structure

As stated earlier, the stiffness coefficient of the sliding springs, representing the lateral compaction of the yarns, needs to be characterized via experimentation. However, the stiffness of torsional springs may be characterized analytically for a given fabric. To derive the latter stifness for PWCP, it is presumed that yarn *Y-3* is a straight beam (Figure 9) made of a linear, transversely isotropic, homogeneous material, with an effective flexural rigidity in the longitudinal direction *Q_b_* [17]. The Euler-Bernoulli theory is used to analyze such a hypothesized beam [16,17]. Moreover, as the proposed model takes the effect of tension on the yarns into account, instead of a first-order analysis that is based on the superposition principle and considers the deflection of the beam independent of the applied tension, the second-order analysis [18] is undertaken, which accounts for the effect of tension on the *overall* flexural rigidity of yarns, $\frac{F\;}{Y_{S/2}}$.

When considering the assumptions made, the deflection of the yarn as a beam at its mid-span and under arbitrary vertical force *F* can be written as [18]:

(2) $$Y_{S/2}=\frac{F\;}{2Q_{b}\;{\left(\frac{T}{Q_{b}}\right)}^{\frac{3}{2}}}\left(\frac{S\sqrt{\frac{T}{Q_{b}}}}{2}-\tanh\left(\frac{S\sqrt{\frac{T}{Q_{b}}}}{2}\right)\right)$$

where *T*, $Q_{b}$, and *S* are tension along the yarn, the effective flexural rigidity of yarn in its longitudinal direction, and the length of yarn in the representative PWCP element (Figure 3), respectively.

It is important to note that theoretically, the second order analysis determines the extent of the increase in bending stiffness due to the applied tension in an integrated (continuum) beam. However, yarns are comprised of thousands of filaments. Accordingly, the effective bending rigidity of yarn, $Q_{b}$, where filaments are only in frictional contact with each other and can slide over one another in the dry form, is much less than when the filaments are perfectly jointed (e.g., the yarn is consolidated with a resin) and make an integrated beam. This critical point may be addressed by measuring the flexural rigidity of the dry yarn via the setup explained in Section 4.2.1, which can directly take the discrete nature of the yarns into account (instead of using the yarns’ tensile modulus along with their width and height, as used for integrated beams). However, one point may still be overlooked: an increase in the internal bending rigidity of yarns can occur due to tension; e.g., by considering more interaction and frictional force between filaments. This fact can be accounted for when $Q_{b}$ in Equation (2) becomes a function of tension level.

Now, by equating the deflection of the mid-span of the beam ($Y_{S/2}$), which is caused by the arbitrary force, *F*, to the nodal displacement of the end of the link caused by the same force (OO′ in Figure 9), the equivalent stiffness of the torsional spring is determined as:

(3) $$K_{t}=\frac{2Q_{b}{\left(\frac{T}{Q_{b}}\right)}^{\frac{3}{2}}w^{2}}{\left(\frac{S\sqrt{\frac{T}{Q_{b}}}}{2}\right)-\tanh\left(\frac{S\sqrt{\frac{T}{Q_{b}}}}{2}\right)}\;=\;\frac{2Q_{b}{\left(\frac{T}{Q_{b}}\right)}^{\frac{3}{2}}{\left(w_{0}-\Delta \right)}^{2}}{\left(\frac{S\sqrt{\frac{T}{Q_{b}}}}{2}\right)-\tanh\left(\frac{S\sqrt{\frac{T}{Q_{b}}}}{2}\right)}$$

where *w*, *w*_0_ and Δ are, in turn, the instantaneous width of yarn, initial width of yarn, and compaction of the yarn due to lateral compressive force. For subsequent calculations, it is convenient to define the following lumped parameter notation:

(4) $$A=\;\frac{2Q_{b}{\left(\frac{T}{Q_{b}}\right)}^{\frac{3}{2}}}{\left(\frac{S\sqrt{\frac{T}{Q_{b}}}}{2}\right)-\tanh\left(\frac{S\sqrt{\frac{T}{Q_{b}}}}{2}\right)}$$

### 3.2. Determination of Critical Compressive Force (P_cr_) and Shear Angle at the Onset of Wrinkling

As discussed in Section 2, the onset of wrinkling in the fabric element is coincident with the instant at which a deviation of deformation mode in the PWCPE structure occurs. As such, the methods developed in the field of structural analysis can be borrowed to estimate the onset of deviation in the equilibrium mode (wrinkling) of PWCPE in Figure 6. One of the de facto analytical approaches is the principle of minimum total potential energy. This approach is based on the assumption that the corresponding mechanical system is conservative (with no energy loss). However, there exist two sources of energy loss due to friction in dry fabrics. The first is the energy dissipated through the friction at crossovers. Because this is substantially less than the lateral compaction energy [2], it should not sizably violate the above assumption. The second source of energy dissipation would be the filaments’ sliding within the yarn while it is under lateral compaction. This mechanism would cause an elastoplastic behavior of the yarn in the lateral direction toward the wrinkling onset. However, upon locking, the compaction (lateral stiffness) of individual filaments is the main source of resistance of the yarn against further fabric shearing, and the sliding between filaments would be minimal due to high normal forces.

**Figure 10:**
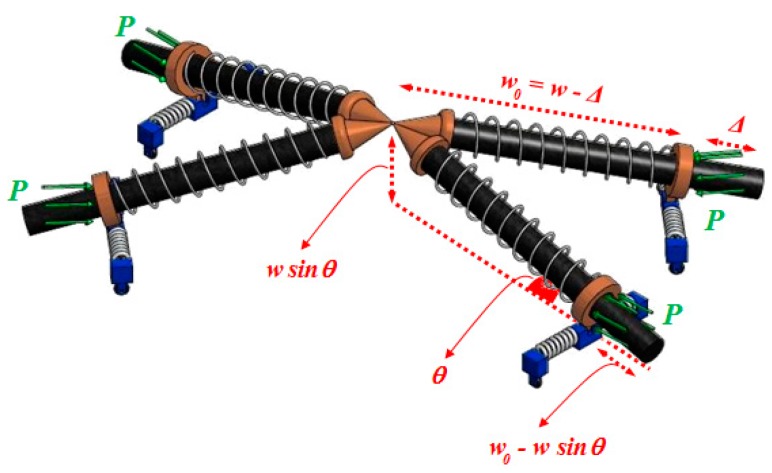
The PWCPE structure in an imaginary deviated mode; note that the *P* forces remain in the original directions.

By taking Figure 10 into consideration, the total potential energy of the system in an imaginary deviated mode is:
(5)
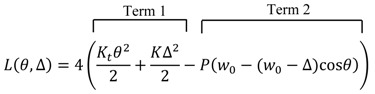

where *θ* is the angle of rods with respect to the plane of initial mode, Δ is the compaction of the slide springs, and *P* is the compressive forces exerted on the sliding springs. In Equation (5), term 1 and term 2 are in turn the strain energy of the system, negative of the work done by the internal forces, and the potential energy of the external forces, negative of work done by external forces [19]. Since the bars in the equivalent structure are assumed as rigid (merely as the cores of the sliding springs), there exists no term of strain energy for these members. Owing to the conservation of energy in the system, the equilibrium of the PWCPE structure in the initial mode is:

(6) $$\{\begin{array}{c} \frac{\partial L}{\partial \theta }=0\;\overset{\;}{\Rightarrow }\;K_{t}\theta -P\left(w_{0}-\Delta \right)\sin\theta =0\;\overset{}{\Rightarrow }\;\theta =0\\ \frac{\partial L}{\partial \Delta }=0\;\overset{}{\Rightarrow }\;K\Delta -P\cos\theta =0\;\overset{}{\Rightarrow }\;\Delta =\frac{P}{K} \end{array}$$

On account of the fact that the PWCPE structure is a system with multiple degrees of freedom, the minimum of total potential energy *L* (*θ*, Δ) can be obtained with the aid of the Hessian matrix. If the matrix is positive definite, then the equilibrium mode of the structure does not intend to deviate to an adjacent equilibrium state—the Lagrange-Dirichlet theorem [20]. However, if the matrix is either negative definite or is not a sign definite, the equilibrium mode of the structure opts to deviate to an adjacent equilibrium mode—the first Lyapunov theorem [20]. Hence, it is inferred that the instant at which the Hessian matrix of the structure changes its sign from positive definite to negative definite, may be considered as the onset of equilibrium mode deviation. The determinant of Hessian matrix for the PWCPE structure is:

(7) $$H=\;{\left|\left[\begin{array}{cc} 4K_{t}-4P\left(w_{0}-\Delta \right)\cos\theta & 4P\sin\theta \\ 4P\sin\theta & 4K \end{array}\right]\right|}_{\begin{array}{l} \theta =0\\ \Delta =\frac{P}{K} \end{array}},$$

The instant at which deviation in deformation mode occurs is defined by *H* = 0. By considering Equations (3) and (4), it is evident that *K_t_* depends on Δ and *A*. Hence, we have:

(8) $$H=\left|\left[\begin{array}{cc} 4A{\left(w_{0}-\Delta \right)}^{2}-4P\left(w_{0}-\Delta \right)\cos\theta & 4P\sin\theta \\ 4P\sin\theta & 4K \end{array}\right]\right|{\;}_{\begin{array}{l} \theta =0\\ \Delta =\frac{P}{K} \end{array}}=0,$$

Substituting Δ by *P*/*K* and *θ* by zero, a quadratic equation with respect to the compressive loads is found, whose roots identify the critical compressive load *P_c_*_r_:

(9) $$\left(1+\frac{A}{K}\right)P^{2}-\left(2Aw_{0}+Kw_{0}\right)P+KAw_{0}^{2}=0,$$

Hence,

(10) $$P_{cr}=\frac{2Aw_{0}+Kw_{0}\pm Kw_{0}}{2\left(1+\frac{A}{K}\right)}$$

Only the minus sign of Equation (10) should be considered since the least critical value of the force is of importance in the instability analysis. Hence, the Hessian matrix changes sign from positive definite to negative definite when:

(11) $$P_{cr}=\frac{AKw_{0}}{A+K}$$

Considering Equation (6), the critical yarn compaction, Δ*_cr_*, can be determined as:

(12) $$\Delta_{cr}=\frac{P_{cr}}{K}=\frac{Aw_{0}}{A+K}=\frac{2Q_{b}{\left(\frac{T}{Q_{b}}\right)}^{\frac{3}{2}}w_{0}}{2Q_{b}{\left(\frac{T}{Q_{b}}\right)}^{\frac{3}{2}}+K\left(\left(\frac{S\sqrt{\frac{T}{Q_{b}}}}{2}\right)-\tanh\left(\frac{S\sqrt{\frac{T}{Q_{b}}}}{2}\right)\right)}$$

Finally, referring to Figure 3 and the discussion presented in Section 2, the shear angle at which wrinkling occurs—the critical shear angle—is:

(13) $$\gamma_{Wrinkling}\;=\cos^{-1}\left(\frac{w_{0}+t-\Delta_{cr}}{l}\right)$$

This implies that as the shear deformation grows, at some point the distance between the adjacent parallel yarns reaches the thickness of the interlacing yarn, where locking begins Equation (1); subsequently, the adjacent parallel yarns start compacting each other up to Δ*_cr_* when eventually wrinkle sets in Equation (13). It should be mentioned that in this analytical model, the compaction of the yarns in the thickness direction, which is substantially less than that in the width direction, is ignored.

## 4. Experimental Evaluation

### 4.1. Geometric Characterization

**Figure 11:**
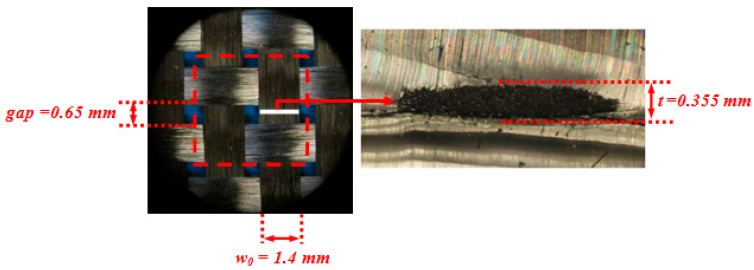
The geometric characteristics of the tested PWCP.

A commercially available PWCP made of carbon fiber was acquired from APC Composite Inc. (Livermore, CA, USA). The geometric specifications of the fabric were obtained using a Nikon optical microscope (Nikon, Tokyo, Japan). For the geometric characterization of yarns, six measurements at different locations of the fabric were made, and the measured values were averaged, as indicated in Figure 11.

To measure the yarn width and the spacing between two adjacent parallel yarns, the PWCP sample was laid under the microscope, and the measurements were taken. However, measuring the thickness of the yarn was not as straightforward. Six yarns were dipped into PDMS, which is a transparent and soft thermoset polymer. After curing the polymer, a section-cut was passed perpendicular to the longitudinal axis of the yarn. Subsequently, the thickness of the yarn was measured under the microscope (Figure 11).

### 4.2. Meso-Mechanical Characterization of the Fabric

Mechanical properties of the yarns comprising flexural rigidity along with lateral compaction stiffness of yarns—in the width direction—needed to be experimentally measured. There are some studies targeting characterization of the effective flexural rigidity of the yarns in the longitudinal direction [17,21], whereas measuring the lateral compaction stiffness of yarns has not received much attention in the literature.

#### 4.2.1. Effective Flexural Rigidity of Yarn in Longitudinal Direction (*Q_b_*)

**Figure 12:**
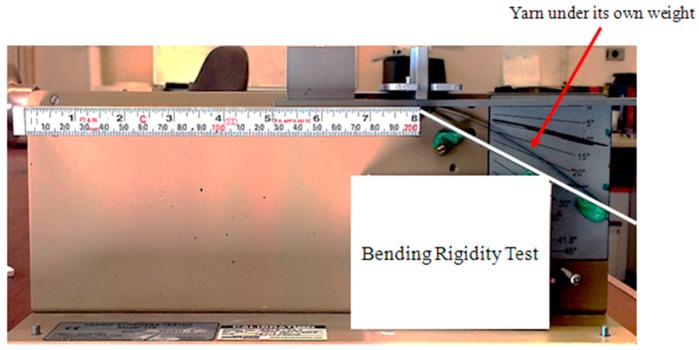
The experimental set-up used for measuring effective bending rigidity of the yarn.

There exists a common test method for determining the effective bending rigidity of a yarn. The experiment is based on Peirce’s cantilever testing procedure, in which a yarn bends under its own weight [17,21]. Figure 12 illustrates the experimental set-up used in this study. Five measurements were taken, and the averaged value for the effective bending rigidity of the yarn was found to be 2.75 N·mm^2^.

#### 4.2.2. Effective Lateral Stiffness of Yarn (*K*)

##### Direct Method

**Figure 13:**
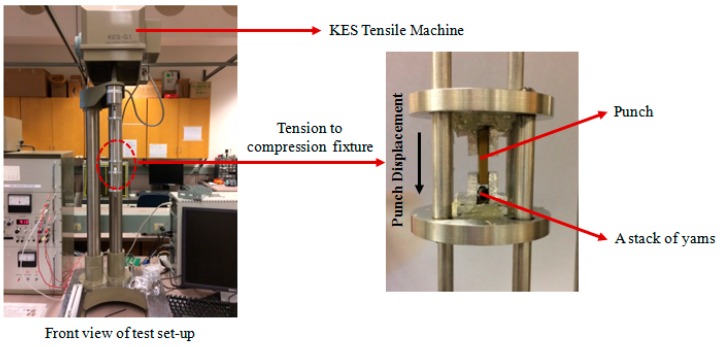
Test set-up used to measure lateral stiffness of the yarn.

The first approach for determining the lateral stiffness of a yarn would be to directly measure the lateral force-lateral displacement of a single yarn—or a stack of yarns—using a compression test, as described in [2]. To do so, an experimental set-up was prepared. An aluminum fixture was designed and fabricated to transform the tensile force provided by a KES-G1 tensile machine (Kato Tech, Kyoto, Japan) to a compressive force. Moreover, a punch, made of brass, was utilized to apply a compressive force to the yarns. Two loose tapes were attached to the yarn stack to mimic the effect of yarn interlacement (Figure 13).

**Figure 14:**
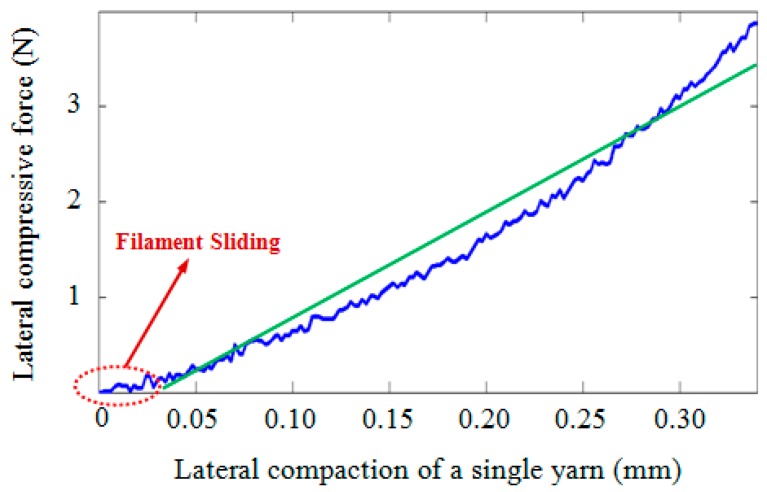
The force-displacement curve for a single yarn under compression in the width direction.

Compressive force was applied to the stack of yarns, and subsequently, the force-displacement curve for a single yarn was extracted based on the procedure given in [2]. Figure 14 shows the force-displacement curve obtained for a single yarn. As can be seen, the graph shows two distinct trends, first of which is related to the elastoplastic behavior of the yarn in which the filaments slide over each other. The amount of energy dissipated by this mechanism is negligible when compared to the second part of the response in which the filaments are laterally under compression. Despite the fact that the second part of the graph has a non-linear behavior with fluctuations, the *R*-squared (coefficient of determination) of the fitted line was found to be 0.93. Hence, for the tested fabric, considering an equivalent linear behavior for the compression of yarn, the slope of the fitted line (10.6 N/mm) was used to estimate *K*.

##### Inverse Method

Another practical approach for determining *K* is to use an inverse method based on a bias extension test. Launay et al. [10] introduced an equation that relates the tensile load to the shear load per unit length (*F_shear_*). By measuring the BET shear load per unit length, the increment of the work done on the fabric element (*dW_shear_*) through shear stress can be obtained as:

(15) $$dW_{shear}=\left(dV\right)\times \tau_{shear}\times \left(d\gamma \right)$$

where *dV* is the volume of fabric element. Hence, the work done on this element from the onset of locking to wrinkling is given by:

(16) $${\left(W_{shear}\right)}_{Locking-to-wrinkling}=S^{2}t\int_{\gamma_{locking}}^{\gamma_{wrinkling}}\tau_{shear}d\gamma =S^{2}\int_{\gamma_{Locking}}^{\gamma_{Wrinkling}}F_{shear}\times d\gamma$$

If we assume that the shear work from locking to wrinkling mainly causes the transverse compaction of the yarns in the fabric element, for the PWCPE we can write:

(17) $${\left(W_{shear}\right)}_{Locking-to-wrinkling}=4\times \left(\frac{1}{2}K\Delta^{2}\right)$$

The value of Δ at wrinkling onset is $\Delta_{cr}$ and can be determined from Equation (13). Then,

(18) $$K=\;\frac{1}{2{\left(w_{0}+t-l\;cos\left(\gamma_{wrinkling}\right)\right)}^{2}}\times \left({\left(W_{shear}\right)}_{locking-to-wrinkling}\right)\;$$

where (*W_shear_*)*_locking-to-wrinkling_* may be obtained from the area under force-displacement of the BET. The value obtained for *K* using this method is 11.53 N/mm. Although the value of *K* extracted using the inverse method is comparable to that of the direct measurement approach, the characterization challenge in the inverse method is deemed to be substantially less, though more subjective. To minimize modeling error, an average value between the direct and inverse approaches was eventually used for the final lateral compaction stiffness of yarn (Table 1). In addition to these two approaches, a micro-level FE simulation may be considered in future studies as a viable alternative.

### 4.3. Bias Extension Test for Validating the Analytical Wrinkle Model

**Figure 15:**
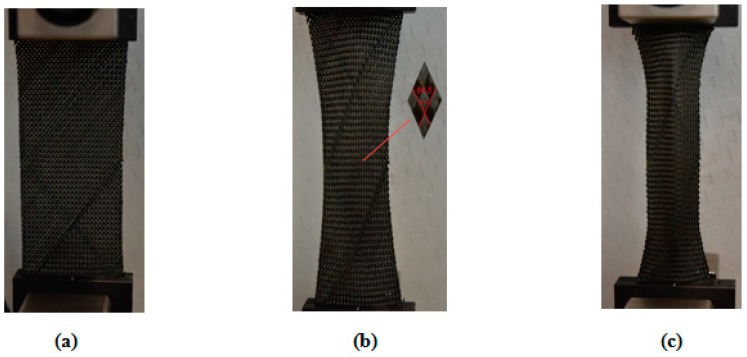
The PWCP (**a**) before shear deformation; (**b**) at the onset of locking (γ=28.7±0.8°); (**c**) at the onset of wrinkling (γ=38±0.5°).

**Figure 16:**
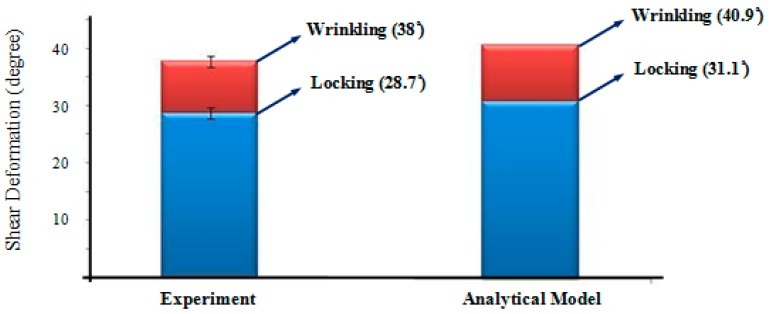
Experimental measurements of locking and wrinkling angles versus the predictions of the proposed analytical model (prediction error for $\gamma_{cr}$ = 9.1%).

By substituting the geometric and mechanical properties of the fabric—as presented in Table 1—into Equations (1) and (13), the locking and critical (wrinkling) shear angles of the woven fabric were determined to be 31.1° and 40.9°, respectively. To evaluate the validity of the proposed equations, a BET was carried out (Figure 15). Three repeats were conducted, and in each repeat, the shear angle of the fabric was recorded using a digital camera mounted in front of the test set-up. Figure 16 shows the values for the measured shear angles corresponding to locking and wrinkling versus those calculated from the analytical approach. The experimental results revealed an acceptable agreement with the analytical predictions, and the difference is expected to decrease further if one relaxes the assumptions made in deriving *K_t_*.

## 5. Concluding Remarks

A mesoscopic study on wrinkling of the PWCPs in the BET was presented based on an instability analysis point of view. By considering the mechanism of wrinkle formation in mesoscale, an equivalent structure mimicking the behavior of a representative PWCP element in the post-locking stage was proposed. The stability of the equivalent structure was assessed using the principle of minimum total potential energy. The onset of instability in the equivalent structure, which is coincident with the onset of wrinkling in the PWCP, was analytically determined. The proposed Equations (12) and (13) imply that a number of parameters influence the extent of the critical shear angle of the PWCPs: the lateral compaction stiffness of yarns (*K*), tension along yarns (*T*), effective flexural rigidity of yarns ($Q_{b}$), initial yarn width (*w*_0_), initial yarn thickness (*t*), the yarn length in the representative PWCP element (*S*), and the initial distance between the central axes of the adjacent parallel yarns (*l*). The analytical model introduced in this paper may be helpful for the following applications.

**Fabric Design for Superior Wrinkling Behavior:** Occurrence of wrinkling in the course of the draping of the PWCPs is currently a major concern and can be of the types illustrated in Figure 1. Among the different types, wrinkling due to large in-plane shear deformation is deemed critical in draping around parts with sharp corners. The presented model may be considered as a first step toward better analytically comprehending the mechanics of wrinkle formation in the PWCPs due to large shear deformation. One of the main merits of the approach is incorporating both the geometric and the mechanical characteristics of the preform. Since the model comprises of mesoscale parameters of the PWCPs, it opens a window toward analyzing the impact of sub-scale contributing parameters on the shear wrinkling behavior of the woven composites. More specifically, upon a parametric study (sensitivity analysis) of the mesoscale specifications of different preforms, optimum PWCPs can be analytically designed/selected to offer superior resistance to wrinkling during forming.

**Minimum Tensioning of Yarns during the Forming Process:** Determining the minimum tension along yarns to evade wrinkling is one of the most practical challenges [22], and the proposed model has the potential to determine this minimum tension. Equation (13) explicitly reveals how the critical shear angle can be affected by adjusting tension along yarns. Referring to Equation (4), it is evident that *A* is increased if tension along yarns *T* is ramped up. By taking Equation (12) into account, Δ*_cr_* becomes larger by the growth of *T*, inferring that wrinkling can be postponed or even fully avoided. Substituting the values of the parameters of a given PWCP and the rough approximation of the expected maximum shear angle into Equation (13), e.g., using available kinematics based draping software, the minimum tension required along yarns to avoid shear wrinkling is obtainable. This cost-effective solution would be useful for the industrial optimization of forming processes of large PWCP parts. In the current study, a linear behavior of yarns under tension was presumed, while for more precise predictions, a model based on a non-linear behavior of the yarns under tension can be developed.

## Figures and Tables

**Table 1:** Summary of the measured geometric and mechanical characteristics of the PWCP.

Yarn Material	Geometric Characteristics				Mechanical Characteristics	
	*w*_0_ (mm)	*S* (mm)	*l* (mm)	*t* (mm)	Flexural Rigidity—$Q_{b}$ (N·mm^2^)	Lateral Stiffness—*K* (N/mm)
Carbon fiber	1.4	4.1	2.05	0.355	2.75	11.06

## Abbreviations

$\gamma_{Locking}$	Locking angle	*P_cr_*	Critical lateral compressive force between yarns
$w_{0}$	Initial yarn width	*Q_b_*	Effective flexural rigidity
t	Yarn thickness	$Y_{S/2}$	Deflection of the yarn as a beam at its mid-span
l	Initial distance between the central axis of the adjacent parallel yarns	*F*	An arbitrary vertical force
*K_t_*	Stiffness of rotation springs	*T*	Tension along the yarn
*K*	Lateral stiffness of yarns	*S*	Length of yarn in the representative PWCP element
*P*	Quasi-static compressive force between yarns	*w*	Instantaneous width of yarn
Δ	Compaction of the yarn due to lateral compressive force	A	Lumped parameter notation
*θ*	Angle of rods with respect to the plane of fabric	L(θ,Δ)	Total potential energy of the system
H	Hessian matrix	$\Delta_{cr}$	Critical yarn compaction
$\gamma_{Wrinkling}$	Wrinkling angle	*x*	Distance from the constraint to any arbitrary point on the yarn
*y*(*x*)	Deflection of the yarn corresponding to *x*	*q*	Weight per length of the yarn
*L’*	Length of the yarn in banding test	*F_shear_*	Shear force
*dW_shear_*	Increment of the work done on the fabric element	$\tau_{shear}$	Shear stress
*dV*	Volume of fabric element		

## References

[1] Chou T.W., Ko F.K. (1988). Textile Structural Composites. Composite Material Series.

[2] Zhu B. (2007). Sheet Forming of Woven Textile Composite Preforms: Formability and Wrinkling. Ph.D. Thesis.

[3] Boisse P., Hamila N., Vidal-Sallé E., Dumont F. (2011). Simulation of wrinkling during textile composite reinforcement forming. Influence of tensile, in-plane shear and bending stiffnesses. Compos. Sci. Technol..

[4] Boisse P., Zouari B., Daniel J.L. (2006). Importance of in-plane shear rigidity in finite element analyses of woven fabric composite preforming. Compos. Part A Appl. Sci. Manuf..

[5] Badel P., Vidal-Sallé E., Boisse P. (2007). Computational determination of in-plane shear mechanical behaviour of textile composite reinforcements. Comput. Mater. Sci..

[6] Kashani M.H., Hosseini A., Sassani F., Ko F.K., Milani A.S. (2017). The Role of Intra-Yarn Shear in Integrated Multi-Scale Deformation Analyses of Woven Fabrics: A Critical Review. Crit. Rev. Solid State Mater. Sci..

[7] Boisse P., Hamila N., Guzman-Maldonado E., Madeo A., Hivet G., Dell’Isola F. (2017). The bias-extension test for the analysis of in-plane shear properties of textile composite reinforcements and prepregs: A review. Int. J. Mater. Form..

[8] Prodromou A.G., Chen J. (1997). On the relationship between shear angle and wrinkling of textile composite preforms. Compos. Part A Appl. Sci. Manuf..

[9] Lightfoot J.S., Wisnom M.R., Potter K. (2013). Defects in woven preforms: Formation mechanisms and the effects of laminate design and layup protocol. Compos. Part A Appl. Sci. Manuf..

[10] Launay J., Hivet G., Duong A.V., Boisse P. (2008). Experimental analysis of the influence of tensions on in plane shear behaviour of woven composite reinforcements. Compos. Sci. Technol..

[11] Zhu B., Yu T.X., Teng J., Tao X.M. (2008). Theoretical modeling of large shear deformation and wrinkling of plain woven composite. J. Compos. Mater..

[12] Zhu B., Yu T.X., Zhang H., Tao X.M. (2008). Experimental investigation of formability of woven textile composite preform in stamping operation. Int. J. Mater. Form..

[13] Liu L., Chen J., Li X., Sherwood J. (2005). Two-dimensional macro-mechanics shear models of woven fabrics. Compos. Part A Appl. Sci. Manuf..

[14] Kaynia N. (2016). Instability-Induced Transformation of Interfacial Layers in Composites and Its Multifunctional Applications. Ph.D. Disseration.

[15] Gambhir M.L. (2004). Stability Analysis and Design of Structures.

[16] Zhu B., Yu T.X., Tao X.M. (2007). An experimental study of in-plane large shear deformation of woven fabric composite. Compos. Sci. Technol..

[17] Cornelissen B., Akkerman R. Analysis of yarn bending behaviour. Proceedings of the 17th International Conference on Composite Materials.

[18] Zamani A.R., Oyadiji S.O. (2009). Analytical modelling of Kirschner wires in Ilizarov circular external fixator as pretensioned slender beams. J. R. Soc. Interface.

[19] Jones R.M. (2006). Buckling of Bars, Plates and Shells.

[20] Perelmuter A.V., Slivker V. (2013). Handbook of Mechanical Stability in Engineering.

[21] Syerko E., Comas-Cardona S., Binetruy C. (2012). Models of mechanical properties/behavior of dry fibrous materials at various scales in bending and tension: A review. Compos. Part A Appl. Sci. Manuf..

[22] Haghi Kashani M., Hosseini A., Sassani F., Ko F.K., Milani A. (2017). Understanding Different Types of Coupling in Mechanical Behavior of Woven Fabric Reinforcement: A Critical Review and Analysis. Compos. Struct..

